# Influence of medical educational background on the diagnostic quality of ChatGPT‐4 responses in internal medicine: A pilot study

**DOI:** 10.1111/eci.70113

**Published:** 2025-09-08

**Authors:** Nicolò Gilardi, Massimo Ballabio, Francesco Ravera, Lorenzo Ferrando, Mario Stabile, Andrea Bellodi, Giovanni Talerico, Benedetta Cigolini, Carlo Genova, Federico Carbone, Fabrizio Montecucco, Christian Bracco, Alberto Ballestrero, Gabriele Zoppoli

**Affiliations:** ^1^ Department of Internal Medicine and Medical Specialties (DiMI) Università degli Studi di Genova Genoa Italy; ^2^ IRCCS Policlinico San Martino Genoa Italy; ^3^ Internal Medicine, Azienda Ospedaliera San Giovanni Addolorata Rome Italy; ^4^ First Clinic of Internal Medicine, Department of Internal Medicine University of Genoa Genoa Italy; ^5^ IRCCS Ospedale Policlinico San Martino, Genoa‐Italian Cardiovascular Network Genoa Italy; ^6^ S.C Medicina Interna AO S. Croce e Carle Cuneo Italy

**Keywords:** artificial intelligence, ChatGPT‐4, clinical decision making, diagnostic ranking, internal medicine, large language models

## Abstract

This pilot study evaluated the influence of medical background on the diagnostic quality of ChatGPT‐4's responses in Internal Medicine. Third‐year students, residents and specialists summarised five complex NEJM clinical cases before querying ChatGPT‐4. Diagnostic ranking, assessed by independent experts, revealed that residents significantly outperformed students (OR 2.33, *p* = .007); though overall performance was low. These findings indicate that user expertise and concise case summaries are critical for optimising AI diagnostics, highlighting the need for enhanced AI training and user interaction strategies.

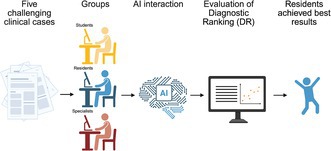

## 1 BACKGROUND

Artificial intelligence (AI) is increasingly being integrated into healthcare, yet its application in internal medicine (IM) remains underexplored. Although some clinical decision support systems—such as INTERNIST‐1 and DXplain—were developed decades ago, they never achieved widespread clinical adoption.^1^, ^2^, ^3^, ^4^ Large language models (LLMs), such as ChatGPT‐4, can generate contextually relevant responses and hold promise as diagnostic aids.^5^, ^6^, ^7^, ^8^, ^9^, ^10^ However, their performance may vary depending on the quality of the input provided by the user. In a future scenario where AI tools might be widely accessible, it is critical to understand whether a user's medical educational background influences diagnostic outputs. Here, we present a pilot study evaluating how varying levels of medical expertise—from third‐year medical students to experienced specialists—affect the quality of ChatGPT‐4's responses to complex IM cases. To achieve this, we developed a novel scoring system to assess ChatGPT‐4's responses quality and to explore how these outputs differ according to the presenter's educational background.

## 2 METHODS

**FIGURE 1:**
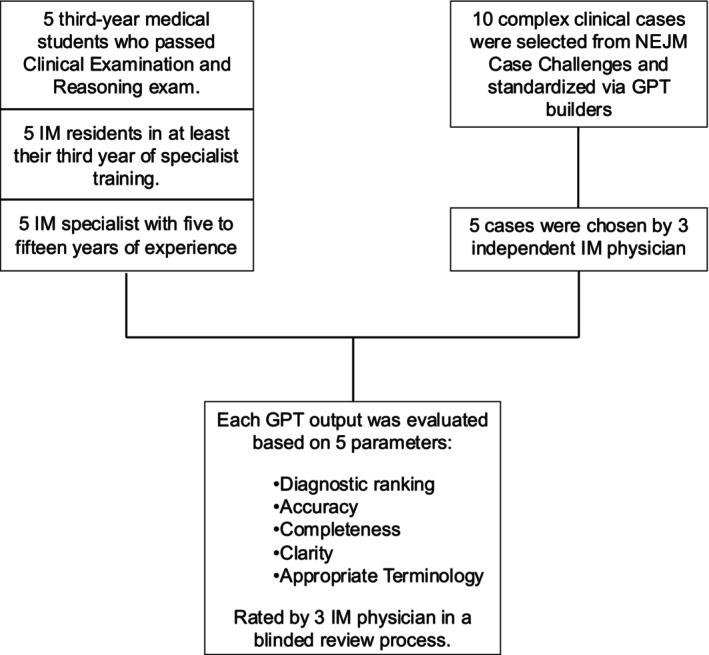
Study design flowchart.

Five complex clinical cases were selected from NEJM Case Challenges based on challenging comorbidities, atypical presentations or rare conditions. Two custom ChatGPT‐4 builders were used to translate the cases into Italian: one retold the clinical history in clear, first‐person, accessible language; the other rendered medical examinations and reports with strict adherence to technical terminology (the Builders are available at the following links: https://chatgpt.com/g/g‐I8ja3pn1E‐clinical‐storyteller; https://chatgpt.com/g/g‐UwrY6VQtW‐clinical‐case‐translator). All cases were stripped of titles, differential diagnosis lists and final diagnoses. An explanatory flowchart of the study design is provided in Figure 1. These clinical cases were presented to 15 participants (enrolled from February 1st to March 31st, 2024) belonging to three groups of varying levels of medical education: (a) third‐year medical students who had passed the Clinical Examination and Reasoning exam; (b) residents in at least their third year of specialist residency in IM; and (c) IM specialists with 5–15 years of consultancy experience.

Participants were instructed to read and summarise each case and then present it to ChatGPT‐4 ‘as if discussing it with a colleague.’ An investigator appended a standardized prompt—‘Based on all the information I have provided to you for this clinical case, give me the top five most likely diagnoses, in decreasing order of probability’—to every case summary. Once obtained, three independent expert IM physicians (not involved in the selection of the cases nor selected as study participants) scored each ChatGPT‐4 response using a novel five‐item system:

1. Diagnostic ranking (DR) (0–10 points): Assessment of the correct diagnosis placement in ChatGPT‐4's responses.
2. Diagnostic strategy and investigation (DS&I) proposal accuracy (0–5 points): Assessment of the logical sequence and medical appropriateness of ChatGPT‐4's suggested investigative strategy, if any.
3. Completeness (0–5 points): Measurement of how comprehensively ChatGPT‐4 addresses the presented clinical case aspects.
4. Clarity and understandability (0–5 points): Evaluation of the clarity and accessibility of ChatGPT‐4's communication.
5. Appropriate use of medical terminology (0–5 points): Assessment of the accuracy and appropriateness of the medical terminology used by ChatGPT‐4.

The original Excel version is available in Appendix S1.

Each response could earn up to 30 points, with detailed scoring criteria provided in Appendix S1.

### 2.1 Statistical analysis

All analyses were conducted in R (v4.4) with data imported via *readxl* and tidied using the *tidyverse* package. Three raters evaluated clinical cases on diagnostic ranking, DS&I proposal accuracy, completeness, clarity, appropriate terminology and an overall score, resulting from the sum of other variables. The merged dataset was pivoted to wide format to enable inter‐rater reliability assessment via intraclass correlation coefficients (ICCs) using a two‐way consistency model from the *irr* package. We selected ICC as our primary reliability metric because it quantifies agreement among multiple raters on continuous measures by partitioning variance between subjects and raters. Additionally, ICC is widely recognised for evaluating subjective clinical assessments.

**FIGURE 2:**
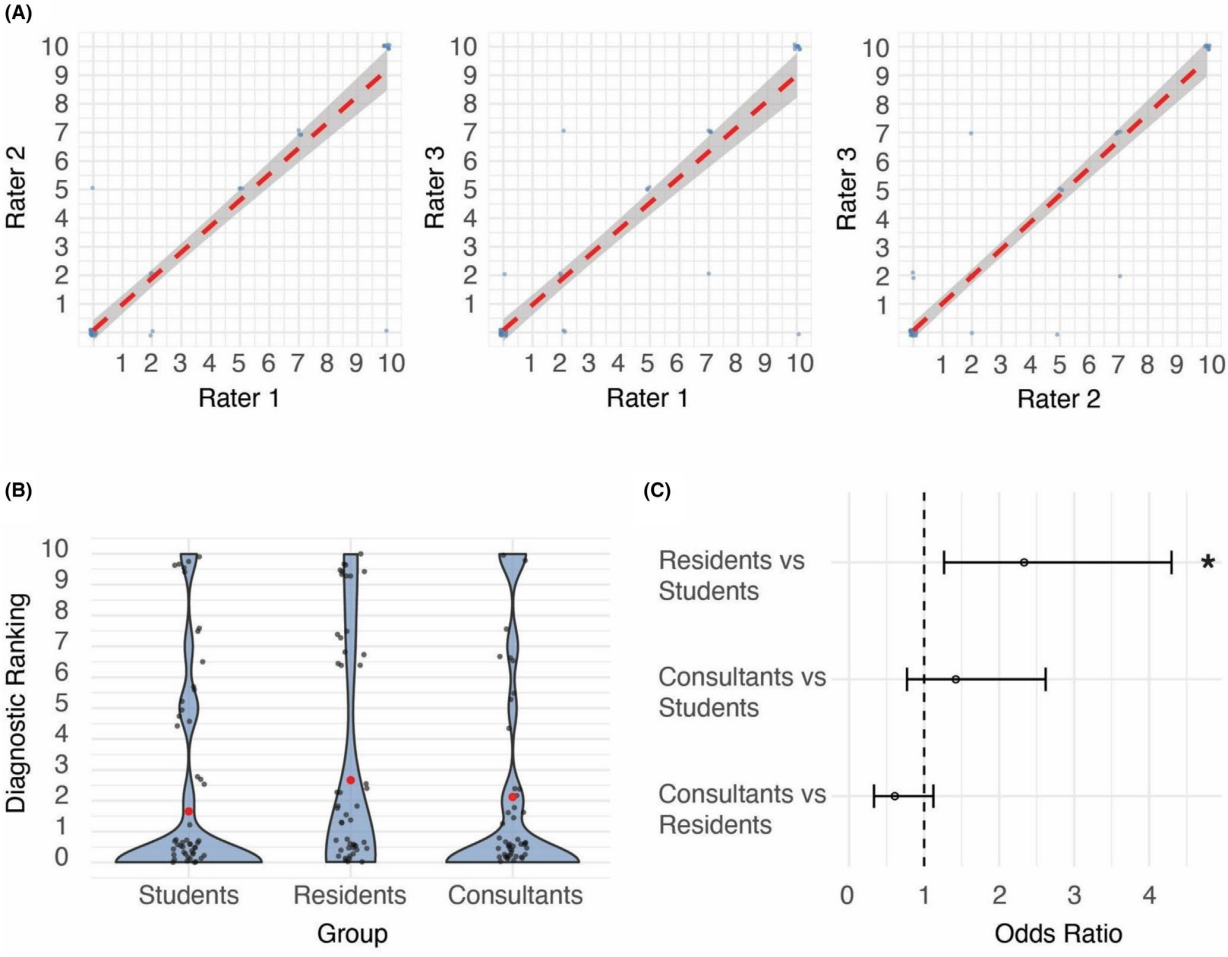
Interrater agreement and study results. (A) Scatter plots of two‐by‐two rater scores for the variable ‘diagnostic ranking’; the red dashed line represents the least square regression, with grey shades highlighting the 95% confidence intervals of the correlations. Spearman's rank coefficients are .91, .91 and .92, respectively. (B) Violin plots of the diagnostic ranking scores according to the evaluating groups; grey dots represent individual diagnostic ranking scores assigned by the independent raters, whereas the thick red dots represent group means. Data distributions are highlighted by the steel blue shades. (C) Forest plot of odds ratios for each represented contrast of groups; *p‐value <.05. Residents versus Students: OR 2.33, 95% confidence interval (CI) = 1.27–4.28, *p*‐value = .007. Consultants versus Students: OR 1.42, 95% CI = .77–2.61, *p*‐value = .258.

Agreement was visualized with Bland–Altman and scatter plots. Diagnostic ranking was normalized using Box–Cox transformations, with optimal lambda determined by log‐likelihood maximization (*MASS* package). Linear mixed‐effects models were fitted (*lme4*, *lmerTest*), with group as a fixed effect and clinical case and rater as random effects; pairwise comparisons were estimated via *emmeans*. Odds ratios with confidence intervals were derived from model contrasts and visualized as a forest plot. Finally, group differences were illustrated using violin plots with jittered points and summary statistics, all saved as PDF figures (Figure 2).

## 3 RESULTS

Demographics of study participants are reported in Table S1. Each rater evaluated every ChatGPT‐4 output using the predefined scoring system (complete data available in Table S1). Notably, only Diagnostic Ranking (DR) demonstrated excellent reliability (ICC = .98), as defined by Koo and Li.^11^ In contrast, Clarity and Appropriate Terminology showed poor reliability, with ICC values of .39 and .15, respectively, suggesting significant variability in how raters assessed these dimensions. Accuracy and Completeness exhibited moderate reliability, with ICC of .52 and .54, respectively, hence indicating some level of agreement, but not to the extent seen with DR. To further assess the discrepancy in results' reliability, the ICC for pairs of raters was calculated, maintaining an ICC greater than .9 for all rater pairs (Figure 1, panel A; Table S2). Such findings underscore the robustness of DR as a reliable metric for evaluating ChatGPT‐4's performance in the presented scenario and prompted us to select it as the comparison parameter for downstream analyses.

DR was therefore selected as the criteria for evaluating study groups' performance (Figure 2, panel B and C). Residents obtained a significantly higher score compared to third‐year students (odds ratio (OR) = 2.33, 95% confidence interval (CI) = 1.27–4.28, *p*‐value = .007). There was a similar trend for specialists, but it did not reach statistical significance (OR = 1.42, 95% CI = .77–2.61, *p*‐value = .258). Number and percent of correct diagnoses by ranking within groups and globally are reported in Table S3. Notably, ChatGPT‐4 performance was low in all study groups.

Taken together, these findings suggest that the medical background of an AI interlocutor significantly influences AI's performance in evaluating complex clinical cases.

## 4 DISCUSSION

In recent decades, AI has become increasingly integrated into medicine, with notable advancements in oncology, radiology and cardiology.^12^ However, its application in IM remains relatively under‐explored. LLMs, such as ChatGPT‐4, hold promise for filling this gap. We evaluate how a user's medical background influences ChatGPT‐4's diagnostic reliability and response quality, driven by concerns of a future scenario where limited public health funds and the widespread availability of low‐cost AI tools might encourage patients to favour automated advice over professional consultation. Previous studies have assessed AI performance in focus domains—like emergency and infectious diseases—or against simulated cohorts. In contrast, our pilot study examined ChatGPT‐4's real‐world performance across five complex IM cases drawn from NEJM Case Challenges, specifically examining how the user's medical expertise influences the quality of its diagnostic output.

Participants ranging from third‐year medical students to experienced specialists summarised each case before querying the model. We then scored responses on five dimensions—Diagnostic Ranking, Accuracy, Completeness, Clarity and Appropriate Terminology—and found that only Diagnostic Ranking demonstrated excellent inter‐rater reliability, validating its use as our principal metric.

This suggests that when querying LLMs about clinical cases, the diagnostic classification serves as a robust framework for evaluating performance. Our results, in contrast to previous studies, revealed a very poor diagnostic performance of ChatGPT‐4 when addressing complex clinical cases, whether interviewed by users with limited medical knowledge or by healthcare professionals (Table S3). In previously published studies, ChatGPT's performance was primarily evaluated in specialised settings or fixed scenarios (e.g. antimicrobial treatment selection, emergency triage and rheumatic diseases). In our study, the presence of multimorbidity, confounding clinical data and the rarity of certain conditions—typical of internal medicine—likely contributed to the challenges faced by this LLM, which is not specifically trained in the medical domain, resulting in poorer diagnostic performance. For one out of five clinical cases, ChatGPT never mentioned the correct diagnosis in its diagnostic list, and in another case, the correct diagnosis was reached in only one out of 15 instances. Interestingly, when we focused on the cases where GPT successfully identified the correct diagnosis as the first or second option, we found that the participants who received the correct diagnosis generally provided concise, focused summaries. They seemed to isolate the most relevant clinical information and/or already had a strong suspicion of the correct diagnosis. In contrast, for the same cases, participants whose GPT responses were incorrect tended to submit longer, less targeted summaries that did not emphasise the critical clinical details. Moreover, the study showed a significant variability in ChatGPT‐4's diagnostic performance depending on the user's medical knowledge, with residents, but not experienced specialists, significantly outperforming medical students. This suggests a ‘digital divide’ in the medical field, where residents, due to their recent training and exposure to technology, are better equipped to leverage AI tools effectively. Our findings underscore the importance of a strong medical knowledge base, conscientious use of AI and a solid foundation in information technology to maximise the performance and effectiveness of AI tools. Used appropriately, AI models like ChatGPT‐4 offer the potential to expedite diagnoses, reduce healthcare costs and ultimately improve patient outcomes. However, they are not yet suitable for independent use by nonexperts, as there is a real risk of replacing essential medical consultation with online tools. Looking ahead, we envision a healthcare landscape where AI systems are seamlessly integrated into digital platforms to speed up diagnostic processes, shorten hospital stays, avoid unnecessary tests and alleviate administrative burdens. Achieving these objectives will require close collaboration among healthcare professionals, AI engineers and governmental agencies to develop robust, user‐friendly solutions that uphold patient safety and clinical effectiveness. Building such trust hinges on transparency. In this regard, Explainable AI seeks to provide transparent ‘why’ and ‘how’ explanations for model outputs, demystifying the decision process and strengthening clinician confidence. Another promising frontier is Graph Neural Networks (GNNs), which represent patients or clinical variables as graph nodes and their interrelations as edges. By aggregating information across these connections, GNNs can uncover latent patterns in high‐dimensional, relational datasets—ideal for the complexity of IM cases.

Our study has several limitations, including a small sample size of participants from a single institution. Increasing the sample size and incorporating participants from diverse backgrounds would enhance the validity and generalisability of our findings. Additionally, we did not consider participants' prior experience with ChatGPT, which could influence their interactions with the model; the 40% of specialists (2 out of 5) never used AI tools before, while among students and residents, GPT was quite well known but little used. However, this approach reflects a realistic scenario where users may have varying levels of familiarity with AI tools. Although AI in IM is not yet widely used, there are several important ethical aspects to consider. First, the use of LLMs by individuals with limited or no medical background poses significant risks. Such users may be unable to provide medically accurate information as input and may misinterpret AI outputs, lacking the ability to recognise possible errors or limitations in the model's responses. Second, both medical and nonmedical users need clear guidelines on how to interact with AI tools. For safe and effective integration of AI models into IM clinical practice, it is crucial that residents and specialists develop and refine the necessary skills to leverage AI responsibly. Finally, broad public education is essential to highlight both the potential benefits and, especially, the risks associated with unregulated or uninformed use of LLMs in healthcare. Such efforts will be decisive in ensuring that AI tools are employed ethically, with appropriate oversight and an understanding of their limitations.

## 5 CONCLUSIONS

Our study highlights the potential and limitations of AI models like GPT‐4 in assisting with clinical decision‐making in IM. The variability in performance based on user expertise underscores the need for continued research and development to optimise AI's role in healthcare. The overall low performance of ChatGPT‐4 highlights the current inadequacy of LLMs in AI‐assisted medical decision‐making in IM. Future studies should explore the continuous engagement of further iterations of GPT with complex clinical cases, including determining diagnostic pathways and responding to clinical test results. Ultimately, AI is not intended to replace physicians but to serve as a valuable tool, enhancing diagnostic accuracy and efficiency, potentially leading to improved patient care and better resource utilisation in healthcare settings.

## AUTHOR CONTRIBUTIONS

NG, MB, GZ and AlB were involved in conceptualization. NG, MB, FR, LF and GZ were involved in methodology. NG, MB, MS, AB, GT, BC, CG, FC, FM and CB were involved in data collection. GZ, LF, FR, NG, MB, CB and FM were involved in data analysis. NG, MB, FR and GZ were involved in writing original draft. All authors were involved in writing review and editing. GZ, FR and LF were involved in visualization. GZ, FR and AlB were involved in supervision. NG and GZ were involved in project administration. AlB and GZ were involved in funding acquisition.

## CONFLICT OF INTEREST STATEMENT

Two study co‐authors of this manuscript are members of the Editorial Board of European Journal of Clinical Investigation. Fabrizio Montecucco is Editor in Chief of European Journal of Clinical Investigation and a co‐author of this article; Federico Carbone is an Editorial Board member of European Journal of Clinical Investigation and a co‐author of this article. To minimize bias, they were excluded from all editorial decision‐making related to the acceptance of this article for publication. The remaining authors declare no significant conflict of interest with this study.

## Supporting information


Appendix S1.

Appendix S2.

## Data Availability

Raw data along with R codes are available upon reasonable request to the corresponding author Gabriele Zoppoli.
