# Supplementary material for: Influence of medical educational background on the diagnostic quality of ChatGPT‐4 responses in internal medicine: A pilot study
Source: Eur J Clin Invest. 2025 Sep 8;55(11):e70113. doi: 10.1111/eci.70113 (PMC12517243; doi:10.1111/eci.70113)
Supplement: Supplementary file 2 — Appendix S2. [file ECI-55-e70113-s001.docx]

**SUPPLEMENTARY DATA**

Summary

[Table S1: Residents’ demographic data 2](#_Toc185159963)

[Table S2: ICC between raters for each parameter 3](#_Toc185159964)

[Table S3: number and percentage of correct diagnosis performed by ChatGPT-4 3](#_Toc185159965)

[GPT4 Builders instructions 4](#_Toc185159966)

***Table S1: Residents’ demographic data***

| ***ID*** | ***Sex*** | ***Year of training*** | ***Age*** | ***Previous use of GPT*** | ***Median age*** |
| --- | --- | --- | --- | --- | --- |
| **Students** | | | |  | **21.8** |
| ***AI_1*** | M | 3 | 21 | Yes |  |
| ***AI_2*** | M | 3 | 22 | No |  |
| ***AI_4*** | F | 3 | 21 | No |  |
| ***AI_6*** | M | 3 | 21 | Yes |  |
| ***AI_11*** | M | 3 | 21 | Yes |  |
| **Residents** | | | |  | **31.6** |
| ***AI_3*** | M | 4 | 30 | No |  |
| ***AI_9*** | F | 4 | 29 | Yes |  |
| ***AI_12*** | M | 3 | 36 | Yes |  |
| ***AI_13*** | F | 3 | 31 | Yes |  |
| ***AI_15*** | F | 4 | 32 | Yes |  |
| **Specialists** | | | |  | **43.4** |
| ***AI_5*** | F | 11 | 42 | No |  |
| ***AI_7*** | M | 9 | 40 | Yes |  |
| ***AI_8*** | M | 14 | 45 | No |  |
| ***AI_10*** | F | 17 | 48 | No |  |
| ***AI_14*** | M | 11 | 42 | Yes |  |

# ***Table S2: ICC between raters for each parameter***

|  | ***Rater 1 vs 2*** | ***Rater 1 vs 3*** | ***Rater 2 vs 3*** | ***Overall DR*** |
| --- | --- | --- | --- | --- |
| ***DIAGNOSTIC RANKING*** | 0.93 | 0.91 | 0.95 | 0.98 |
| ***ACCURACY*** | 0.28 | 0.36 | 0.16 | 0.52 |
| ***COMPLETENESS*** | 0.31 | 0.34 | 0.21 | 0.54 |
| ***CLARITY*** | 0.10 | 0.09 | 0.30 | 0.39 |
| ***APPROPRIATE TERMINOLOGY*** | 0.11 | - 0.08 | 0.16 | 0.15 |
| ***TOTAL*** | 0.82 | 0.69 | 0.77 | 0.90 |

# ***Table S3: number and percentage of correct diagnosis performed by ChatGPT-4***

|  | ***First diagnosis*** | ***Second diagnosis*** | ***Third diagnosis*** | ***Fourth or fifth diagnosis*** | ***Out of ranking*** |  |
| --- | --- | --- | --- | --- | --- | --- |
| ***3rd year students **** | *2 (8%)* | *1 (4%)* | *2 (8%)* | *1 (4%)* | *19 (76%)* |  |
| ***Residents **** | *4 (16%)* | *3 (12%)* | *0* | *4 (16%)* | *14 (56%)* |  |
| ***Specialist **** | *4 (16%)* | *1 (4%)* | *1 (4%)* | *4 (16%)* | *15 (60%)* |  |
| ***Total out of 75 cases ***** | *10 (13%)* | *5 (6%)* | *3 (4%)* | *9 (12%)* | *48 (64%)* |  |

** Percentage is calculated on a total of 25 clinical cases (i.e. the total per group)*

*** Percentage is calculated on a total of 75 clinical cases (i.e. 25 cases per group)*

*In this study, the same five clinical cases were submitted to each participant. Each participant created a personalized summary of each case and then presented it to ChatGPT-4—treated as a “professional consultant”—requesting its top five most likely diagnoses. We evaluated ChatGPT-4’s responses using our scoring system, with* ***Diagnostic Ranking (DR)*** *as the primary metric of interest. Specifically, we recorded how frequently ChatGPT-4 identified the correct diagnosis as the first, second, third, fourth/fifth, or out of the top five positions (i.e., “Out of Ranking”).*

***Table S3*** *shows the number and percentage of correct diagnoses for each group of participants (third-year medical students, residents, and specialists) as well as the aggregate results.*

*Overall, these data indicate that ChatGPT-4 demonstrated relatively low diagnostic accuracy in this specific setting, with most correct diagnoses either ranked too low or not included in the top five suggestions at all. However, the differences between groups suggest that medical knowledge and the ability to prompt or “dialogue” with the AI effectively may enhance its diagnostic performance. Consequently, while ChatGPT-4 can offer useful insights, it should be seen as an adjunct rather than a substitute for expert clinical judgment, particularly in complex internal medicine scenarios.*

# ***GPT4 Builders instructions***

*First Builder:*

*The GPT is designed to transform a clinical case described in the third person and retell that case from the first-person perspective, using clear, straightforward Italian, with a direct tone, with mild concern and avoiding medical terms of medium and high complexity. The language of input and output had to be in Italian language. This approach aims to make the information more accessible and relatable. The GPT should ensure the story maintains the essential facts and context of the original case, while adjusting the narrative style to be engaging and understandable to a general audience. The transformation process involves creative writing skills, with a focus on empathy and accessibility. It should avoid any form of medical advice or diagnosis, sticking strictly to storytelling.” (https://chatgpt.com/g/g-I8ja3pn1E-clinical-storyteller)*

*Second Builder:*

- *Role and Goal: This GPT is a standardized translator for medical clinical cases, designed to provide detailed, direct, and medically appropriate translations from English to Italian without personal interpretation. It is specifically tailored to translate medical examinations and investigation reports, including CT scans, MRIs, etc., with high precision and special attention to specific or less common medical terminology.*
- *Constraints: The GPT should avoid personal interpretations or opinions in the translations, ensuring that medical terminology, particularly those used in examinations and reports, is accurately translated in line with professional medical standards.*
- *Guidelines: For each translation request, the GPT should focus on precise and accurate translation, prioritizing medical terms and phrases used in examinations and reports. It should request clarification only when the input lacks necessary detail for an accurate translation or is ambiguous.*
- *Clarification: The GPT should bias towards making a response based on intended behavior, filling in any missing details by assuming a standard medical context. However, it will seek clarification if provided information is too vague or missing crucial details for the accurate translation of examination and report terminology.*
- *Personalization: The GPT will maintain a professional tone, focusing on the technical and medical accuracy of translations, especially those involving complex examination and report terminology.” (https://chatgpt.com/g/g-UwrY6VQtW-clinical-case-translator)*
